# Coping Strategies in the Spanish Population: The Role in Consequences of COVID-19 on Mental Health

**DOI:** 10.3389/fpsyt.2021.606621

**Published:** 2021-04-29

**Authors:** María del Mar Molero Jurado, María del Carmen Pérez-Fuentes, Elena Fernández-Martínez, África Martos Martínez, José Jesús Gázquez Linares

**Affiliations:** ^1^Department of Psychology, Faculty of Psychology, University of Almería, Almería, Spain; ^2^Department of Nursing and Physiotherapy, University of León, León, Spain; ^3^Department of Psychology, Faculty of Psychology, Universidad Autónoma de Chile, Santiago, Chile

**Keywords:** coping, health, COVID-19, adult population, well-being

## Abstract

The worldwide health emergency caused by COVID-19 is a new challenge for humanity which individuals respond to in a diversity of ways. The type of coping people use in such a situation could lead to positive or negative consequences to their health. Our objective was to analyze the use of coping strategies in the general population with attention to sociodemographic variables, and to test the capacity of these strategies for mediating in repercussions on mental health. The 1,160 adults who participated in this study answered the Cognitive Emotion Regulation Questionnaire (CERQ-S) and General Health Questionnaire (GHQ-28). The data were collected in a CAWI (Computer Aided Web Interviewing). The results suggest that the coping strategies they used the most differed depending on sociodemographic characteristics, such as age, sex and education. Furthermore, two mediation models were estimated for positive and negative coping strategies in the relationship between the presence of COVID-19 near them and mental health. The “negative” coping strategies were found to exert an indirect effect as mediators in the impact that COVID-19 positive cases near them had on their health. The consequences to mental health of the impact of coping with adverse situations should not be underestimated and it is important to design programs to educate the population in coping strategies that promote their health.

## Introduction

COVID-19 has led to a worldwide health crisis without precedent. The World Health Organization (WHO) declared it a global emergency on January 30, 2020 (1). Beyond the tensions inherent to the disease itself, the governmental instructions on mass home confinement are a new situation for the Spanish population and generate concern for how people will react, and the repercussions on their mental health this could lead to. A recent review on psychological effects in samples of people in quarantine revealed associated confusion, boredom, insomnia, stress, irritability and depression, some of which continued after it was over (2). Another study by Pérez-Fuentes et al. (3) in an adult Spanish population showed that confinement brought negative consequences to their well-being and negative affect increased both perception of threat from COVID-19 and negative mood, which in turn, increased somatic complaints (4). During the pandemic, health problems were more frequent in young people and singles (5, 6).

During adverse situations threatening well-being, such as the COVID-19 pandemic we are now going through, people use their psychological resources to cope with the situation, developing different styles and strategies. Coping can be considered an effort to reduce or eliminate the negative effects of stress on one's well-being (7). Studies have demonstrated that effective coping strategies can protect people from mental illness when faced with adverse situations (5, 8–10). And the opposite is observed with maladaptive coping strategies, which influence their mental health predisposing them to alterations such as depression and anxiety (6, 11–15), so repercussions on well-being depend on the type of coping used (3, 16).

Based on the Threats and Coping Appraisal Theory (17), it may be said that individuals who are exposed to stressful situations respond with adaptive behavior, which provide them with immediate and long-term well-being, or with maladaptive coping, which distracts or alleviates them, making them feel better temporarily, but generating psychological distress later. However, it is not clear how some coping strategies behave in this relationship with health. Adaptive strategies such as positive reevaluation and refocusing in particular do not seem to have a continued effect over time (18, 19).

The gender perspective should not be forgotten. Coping styles can differ by gender. Women use more emotional coping strategies, such as social support, which could prevent depression (20–23). And men use self-distraction and self-blame more than women (22). One of our hypotheses was therefore the presence of differences in coping strategies between men and women in a context of threat from COVID-19. It has also been confirmed that young women caregivers are the group showing the highest stress levels (22) and those who perceive strong threat from COVID-19 (24). Age is a variable which also seems to influence the choice of coping strategies (22, 25, 26) as does education (24). However, no differences in the use of coping strategies by education level were found in the study by Amazue and Onyishi (27). Therefore, the second hypothesis posed is the existence of differences in coping strategies used by age and education.

Another hypothesis tested was the existence of differences in mental health based on coping style used. The use of cognitive and prosocial behaviors was associated with fewer mental health problems (9). Other variables that could be influencing people's well-being is the existence of positive cases of SARS-CoV-2 near them or staying in places where there has been a high incidence of the disease (6, 9). Therefore, it was expected that having someone nearby diagnosed with COVID-19 would affect their mental health, with coping strategies mediating in this relationship.

The main objective of this study was to analyze the use of coping strategies in the general population with attention to sociodemographic variables, and to test the capacity of these strategies to mediate in the repercussion on their mental health.

## Materials and Methods

### Participants

A total of 1,688 adults originally filled in the survey. After a first review, 528 cases were eliminated from the sample either because the survey was incomplete, or because incoherent or random answers were identified.

The final sample was made up of 1,160 adults residing in Spain, with a mean age of 38.29 (SD = 13.71) in a range of 18–82. Of the whole sample, 30.1% (*n* = 349) were men and 69.9% (*n* = 811) women, with a mean of 41.16 (SD = 14.13) and 37.05 (SD = 13.34), respectively. Of these, 47% (*n* = 545) were single and 53% (*n* = 615) were not.

Apart from the above, and in regard to COVID-19, participants were asked whether they had any positive cases near them. The answer of 31% (*n* = 360) of the participants was positive.

### Instruments

The following instruments were used to collect the data:

An *ad hoc* questionnaire was used for collecting sociodemographic characteristics. Items were included for sex, age, marital status and whether anyone near them was COVID-19 positive.

*Cognitive Emotion Regulation Questionnaire* (CERQ) (28), Spanish version (CERQ-S) (29). This consists of 36 items answered on a five-point Likert type scale (from 1 = almost never, to 5 = almost always). It evaluates nine cognitive strategies for coping with negative situations. Reliability found for the sample in this study was: self-blame (ω = 0.71; GLB = 0.73), acceptance (ω = 0.71; GLB = 0.75), rumination (ω = 0.77; GLB = 0.77), positive refocusing (ω = 0.86; GLB = 0.85), planning (ω = 0.80; GLB = 0.82), positive reappraisal (ω = 0.84; GLB = 0.88), putting into perspective (ω = 0.68; GLB = 0.74), catastrophizing (ω = 0.72; GLB = 0.78), and other-blame (ω = 0.90; GLB = 0.91).

*General Health Questionnaire* (GHQ-28) (30), Spanish adaptation validated by Lobo et al. (31). This scale has 28 items with four answer choices which provide information on somatic symptoms, anxiety and insomnia, social dysfunction and depression subscales. Among the scoring methods is a Likert-type scale, where each answer is scored 0–3. The instrument's reliability in our case was ω = 0.93 and GLB = 0.94 for the whole scale, and for the each of the subscales: somatic symptoms (ω = 0.86; GLB = 0.89), anxiety and insomnia (ω = 0.90; GLB = 0.95), social dysfunction (ω = 0.81; GLB = 0.82) and depression (ω = 0.91; GLB = 0.94).

### Procedure

Data were collected in a CAWI (Computer Aided Web Interviewing) interview after snowball sampling, specifically from 1 to 12 May 2020. Participation was voluntary and before starting to answer the questionnaire, on a first page, relevant information on the study and its purpose was provided. The participants gave their informed consent by marking a box for the purpose, which then allowed them to continue with the questionnaire. They were asked to answer sincerely, and were guaranteed the anonymity of their answers. Random or incongruent answers were detected by control questions inserted throughout the questionnaire. This study was approved by the University of Almeria Bioethics Committee (Ref. UALBIO2020/032).

### Data Analysis

The McDonald's Omega coefficient was estimated to examine the reliability of the instruments, following Ventura-León and Caycho (32). The Greatest Lower Bound (GLB) was also calculated.

Then, the *t*-test for independent samples was applied to examine the differences between groups (age, sex, marital status, education, anyone COVID-19 positive nearby) with regard to coping strategies, and Cohen's *d* (33) was used to quantify the effect size. A Pearson's coefficient correlation analysis was performed to test the relationships between the variables, and the descriptive statistics were calculated.

Finally, the various mediation analyses were performed, taking presence of a COVID-19 positive case nearby as the predictor, and coping strategy mediators, and as result variables the health subscales (somatic symptoms, anxiety/insomnia, social dysfunction and depression). JASP version 0.11.1 (34) based on lavaan was used for this (35). Bias-corrected percentile bootstrap confidence intervals were applied as suggested by Biesanz et al. (36).

## Results

### Coping Strategies for Threat From COVID-19: Sociodemographic Variables

First, a negative correlation was found between age and rumination (*r* = −0.23; *p* < 0.001; 95% *CI* −0.17, −0.28). Other correlations with age, although less intense, were observed with acceptance (*r* = −0.07; *p* < 0.05; 95% *CI* −0.01, −0.13) and putting into perspective (*r* = −0.07; *p* < 0.05; 95% *CI* −0.01, −0.12). When the age variable was dichotomized, taking the sample mean of about 40 as the reference, differences were found between the under 40 (or young adults) (54.8%, *n* = 636) and over 40 (or mature adults) (45.2%, *n* = 524) age groups. In particular, statistically significant differences were observed in rumination [*t*_(1, 158)_ = 6.46, *p* < 0.001, *d* = 0.38].

**Figure 1:**
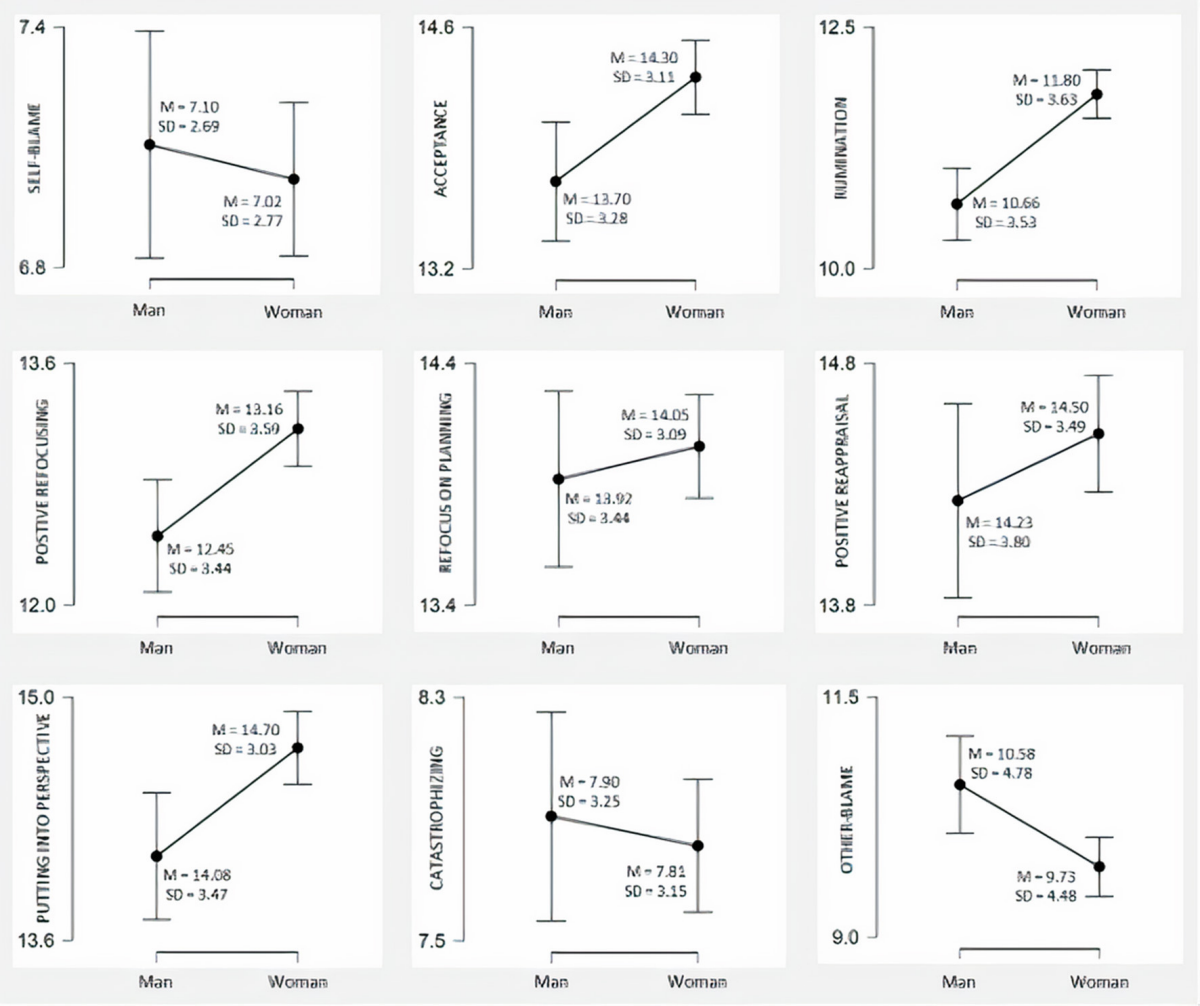
Coping strategies according to sex. Descriptive plots.

Figure 1 shows the results of the comparison of coping strategies by sex. As observed, women scored statistically significantly higher means than men in: acceptance [*t*_(1, 158)_ = −2.97, *p* < 0.01, *d* = −0.19], rumination [*t*_(1, 158)_ = −4.91, *p* < 0.001, *d* = −0.31], positive refocusing [*t*_(1, 158)_ = −3.10, *p* < 0.01, *d* = −0.19], and putting into perspective [*t*_(1, 158)_ = −3.06, *p* < 0.01, *d* = −0.19]; while men scored significantly higher in blaming others [*t*_(1, 158)_ = 2.91, *p* < 0.01, *d* = 0.18].

By marital status at the time of data collection, differences were found between the groups in rumination [*t*_(1, 158)_ = 3.77, *p* < 0.001, *d* = 0.22], where those who did not have a partner scored higher (*M* = 11.88, *SD* = 3.63) than those who had a partner (*M* = 11.08, *SD* = 3.61). No significant differences were observed in the rest of the strategies.

Finally, by education, differences were observed between the primary/secondary education, and higher o university education groups (Table 1). Specifically, differences were found in favor of the group with higher or university studies in the following strategies: rumination, planning, positive reappraisal and putting into perspective. Those with primary/secondary education had significantly higher mean scores in self-blame, catastrophizing and other-blame strategies.

**Table 1:** Coping strategies by education level.

**CERQ**	**Primary/secondary**			**Higher or University education**			***t***	***p***	**Mean Dif**.	**SE Dif**.	**95%** ***CI*** **Mean Dif**.		**Cohen's *d***
	***N***	***M***	***SD***	***N***	***M***	***SD***					**Lower**	**Upper**	
SB	267	7.35	2.86	893	6.95	2.70	2.10	0.036	0.40	0.19	0.02	0.77	0.15
AC	267	13.83	3.33	893	14.21	3.12	−1.68	0.092	−0.37	0.22	−0.80	0.06	−0.12
RU	267	11.03	3.67	893	11.58	3.62	−2.18	0.029	−0.55	0.25	−1.05	−0.05	−0.15
PF	267	12.81	3.63	893	12.99	3.57	−0.69	0.485	−0.17	0.25	−0.66	0.31	−0.05
RP	267	13.58	3.27	893	14.14	3.17	−2.53	0.012	−0.56	0.22	−1.00	−0.12	−0.18
PR	267	13.78	3.77	893	14.61	3.51	−3.32	<0.001	−0.82	0.24	−1.31	−0.34	−0.23
PP	267	14.07	3.19	893	14.65	3.17	−2.60	0.009	−0.57	0.22	−1.01	−0.14	−0.18
CA	267	8.51	3.22	893	7.63	3.14	3.95	<0.001	0.87	0.22	0.44	1.30	0.28
OB	267	10.87	4.73	893	9.72	4.51	3.62	<0.001	1.15	0.31	0.52	1.77	0.25

*CERQ_SB, Self-blame; CERQ_AC, Acceptance; CERQ_RU, Rumination; CERQ_PF, Positive refocusing; CERQ_RP, Refocus on planning; CERQ_PR, Positive reappraisal; CERQ_PP, Putting into perspective; CERQ_CA, Catastrophizing; CERQ_OB, Other-blame*.

*Independent samples t-test*.

### Coping Strategies and Mental Health

Table 2 shows the correlation matrix between coping strategies and the GHQ-28 subscales. Some strategies were positively correlated with the presence of health problems. Rumination and catastrophizing in particular, were positively correlated with all the health subscales, while self-blame and other-blame were positively correlated with the presence of somatic symptoms, anxiety/insomnia and depression. Acceptance was positively correlated, although less intensely, with social dysfunction and depression.

**Table 2:** Coping strategies and mental health: Pearson's correlation matrix and descriptive statistics.

		**GHQ-SS**	**GHQ-AI**	**GHQ-SD**	**GHQ-D**	***M* (*SD*)**
CERQ_SB	*Pearson*′*s*r**	0.083**	0.107***	0.043	0.201***	7.04 (2.74)
	**p**−*value*	0.004	<0.001	0.147	<0.001	
	*Upper*95*%*CI**	0.140	0.164	0.100	0.256	
	*Lower*95*%*CI**	0.026	0.050	−0.015	0.145	
CERQ_AC	*Pearson*′*s*r**	0.046	0.054	0.066*	0.087**	14.12 (3.17)
	**p**−*value*	0.117	0.065	0.026	0.003	
	*Upper*95*%*CI**	0.103	0.111	0.123	0.144	
	*Lower*95*%*CI**	−0.012	−0.003	0.008	0.029	
CERQ_RU	*Pearson*′*s*r**	0.332***	0.438***	0.228***	0.342***	11.46 (3.64)
	**p**−*value*	<0.001	<0.001	<0.001	<0.001	
	*Upper*95*%*CI**	0.383	0.483	0.282	0.392	
	*Lower*95*%*CI**	0.280	0.390	0.173	0.290	
CERQ_PF	*Pearson*′*s*r**	−0.067*	−0.107***	−0.182***	−0.261***	12.95 (3.58)
	**p**−*value*	0.022	<0.001	<0.001	<0.001	
	*Upper*95*%*CI**	−0.010	−0.050	−0.126	−0.207	
	*Lower*95*%*CI**	−0.124	−0.164	−0.237	−0.314	
CERQ_RP	*Pearson*′*s*r**	−0.040	−0.026	−0.121***	−0.140***	14.01 (3.20)
	**p**−*value*	0.176	0.382	<0.001	<0.001	
	*Upper*95*%*CI**	0.018	0.032	−0.064	−0.083	
	*Lower*95*%*CI**	−0.097	−0.083	−0.177	−0.196	
CERQ_PR	*Pearson*′*s*r**	−0.137***	−0.164***	−0.250***	−0.273***	14.42 (3.59)
	**p**−*value*	<0.001	<0.001	<0.001	<0.001	
	*Upper*95*%*CI**	−0.080	−0.108	−0.196	−0.219	
	*Lower*95*%*CI**	−0.193	−0.220	−0.304	−0.325	
CERQ_PP	*Pearson*′*s*r**	−0.034	−0.068*	−0.061*	−0.105***	14.51 (3.18)
	**p**−*value*	0.241	0.020	0.036	<0.001	
	*Upper*95*%*CI**	0.023	−0.011	−0.004	−0.048	
	*Lower*95*%*CI**	−0.092	−0.125	−0.119	−0.162	
CERQ_CA	*Pearson*′*s*r**	0.318***	0.428***	0.215***	0.362***	7.84 (3.18)
	**p**−*value*	<0.001	<0.001	<0.001	<0.001	
	*Upper*95*%*CI**	0.369	0.474	0.270	0.411	
	*Lower*95*%*CI**	0.265	0.380	0.160	0.311	
CERQ_OB	*Pearson*′*s*r**	0.099****	0.124***	0.054	0.100***	9.98 (4.59)
	**p**−*value*	<0.001	<0.001	0.064	<0.001	
	*Upper*95*%*CI**	0.155	0.180	0.112	0.157	
	*Lower*95*%*CI**	0.041	0.067	−0.003	0.043	
	**M**(**SD**)	7.43(4.56)	8.95(5.41)	8.75(3.57)	2.85(4.22)	

CERQ_SB, Self-blame; CERQ_AC, Acceptance; CERQ_RU, Rumination; CERQ_PF, Positive refocusing; CERQ_RP, Refocus on planning; CERQ_PR, Positive reappraisal; CERQ_PP, Putting into perspective; CERQ_CA, Catastrophizing; CERQ_OB, Other-blame. GQ-SS, Somatic symptoms; GHQ-AI, Anxiety/insomnia; GHQ-SD, Social dysfunction; GHQ-D, Depression. ***p < 0.001, **p < 0.01, *p < 0.05.

However, positive refocusing and positive reappraisal correlated negatively with all the GHQ-28 subscales, putting into perspective was related negatively to anxiety/insomnia, social dysfunction and depression, and planning was negatively correlated with social dysfunction and depression.

### COVID-19 Nearby, Coping and Mental Health: Mediation Models

Two mediation models were proposed. In both cases, the predictor was the presence or not of a positive case of COVID-19 nearby, and as the outcome variables, the four GHQ-28 subscales. Model 1, where the mediating effect of “negative” coping strategies (considered as such based on the positive association with the presence of mental health problems) such as rumination and catastrophizing, was hypothesized. Meanwhile, in Model 2, the existence of a mediating effect was hypothesized for the “positive” coping strategies (considered as such based on the negative association found with presence of mental health problems), which were positive refocusing and reappraisal.

In Model 1 (Table 3), a direct effect of positive cases of COVID-19 nearby on the presence of somatic symptoms was observed. As indirect effects, both rumination and catastrophizing mediated in the impact that presence of COVID-19 cases nearby had on health. The total effects were statistically significant for somatic symptoms, anxiety/insomnia and depression.

**Table 3:** Direct, indirect, and total effects (Model 1).

						**95% CI**	
		**Estimate**	**Std. Error**	***z*-value**	***p***	**Lower**	**Upper**
**Direct effects**							
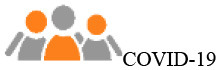	→ GHQ-SS	0.256	0.058	4.404	<0.001	0.135	0.369
	→ GHQ-AI	0.102	0.055	1.862	0.063	−8.21e−4	0.208
	→ GHQ-SD	0.022	0.061	0.365	0.715	−0.102	0.139
	→ GHQ-D	0.066	0.058	1.141	0.254	−0.044	0.210
**Indirect effects**							
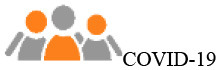	→ CERQ_RU → GHQ-SS	0.032	0.015	2.094	0.036	0.003	0.062
	→ CERQ_CA → GHQ-SS	0.030	0.014	2.122	0.034	0.006	0.062
	→ CERQ_RU → GHQ-AI	0.043	0.020	2.131	0.033	0.004	0.080
	→ CERQ_CA → GHQ-AI	0.041	0.019	2.178	0.029	0.009	0.082
	→ CERQ_RU → GHQ-SD	0.023	0.011	2.010	0.044	0.003	0.046
	→ CERQ_CA → GHQ-SD	0.020	0.010	2.002	0.045	0.003	0.045
	→ CERQ_RU → GHQ-D	0.031	0.015	2.090	0.037	0.003	0.062
	→ CERQ_CA → GHQ-D	0.037	0.017	2.159	0.031	0.008	0.078
**Total effects**							
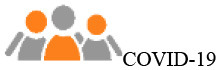	→ GHQ-SS	0.318	0.063	5.073	<0.001	0.195	0.442
	→ GHQ-AI	0.185	0.063	2.934	0.003	0.063	0.305
	→ GHQ-SD	0.065	0.063	1.033	0.302	−0.066	0.188
	→ GHQ-D	0.134	0.063	2.120	0.034	0.017	0.282

*
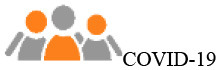
, Anyone COVID-19 positive nearby; CERQ_RU, Rumination; CERQ_CA, Catastrophizing; GHQ-SS, Somatic symptoms; GHQ-AI, Anxiety/insomnia; GHQ-SD, Social dysfunction; GHQ-D, Depression (Delta method standard errors, bias-corrected percentile bootstrap confidence intervals)*.

Model 2 (Table 4) showed significant direct effects of the presence of COVID-19 cases nearby on somatic symptoms and anxiety/insomnia. However, this second proposal was not significant for the indirect effects of positive refocusing and reappraisal as mediators in the relationship between the presence of COVID-19 positives nearby and its impact on health. That is, the use of these strategies did not mediate or buffer the relationship between predictor and outcome variables.

**Table 4:** Direct, indirect, and total effects (Model 2).

						**95% CI**	
		**Estimate**	**Std. Error**	***z*-value**	***p***	**Lower**	**Upper**
**Direct effects**							
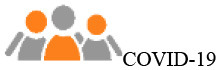	→ GHQ-SS	0.311	0.062	4.996	<0.001	0.199	0.435
	→ GHQ-AI	0.176	0.062	2.815	0.005	0.054	0.301
	→ GHQ-SD	0.050	0.061	0.814	0.416	−0.085	0.174
	→ GHQ-D	0.116	0.060	1.923	0.055	−0.006	0.244
**Indirect effects**							
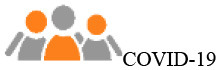	→ CERQ_PF → GHQ-SS	−1.02e−4	0.002	−0.068	0.946	−0.008	0.004
	→ CERQ_PR → GHQ-SS	0.008	0.009	0.880	0.379	−0.006	0.030
	→ CERQ_PF → GHQ-AI	0.002	0.003	0.583	0.560	−0.002	0.015
	→ CERQ_PR → GHQ-AI	0.008	0.009	0.883	0.377	−0.007	0.031
	→ CERQ_PF → GHQ-SD	0.003	0.005	0.684	0.494	−0.004	0.019
	→ CERQ_PR → GHQ-SD	0.012	0.014	0.893	0.372	−0.012	0.043
	→ CERQ_PF → GHQ-D	0.008	0.011	0.708	0.479	−0.012	0.032
	→ CERQ_PR → GHQ-D	0.011	0.012	0.891	0.373	−0.011	0.041
**Total effects**							
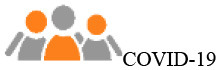	→ GHQ-SS	0.318	0.063	5.073	<0.001	0.207	0.443
	→ GHQ-AI	0.185	0.063	2.934	0.003	0.054	0.308
	→ GHQ-SD	0.065	0.063	1.033	0.302	−0.078	0.189
	→ GHQ-D	0.134	0.063	2.120	0.034	0.018	0.279

*
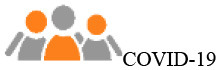
, Anyone COVID-19 positive nearby; CERQ_PF, Positive refocusing; CERQ_PR, Positive reappraisal; GHQ-SS, Somatic symptoms; GHQ-AI, Anxiety/insomnia; GHQ-SD, Social dysfunction; GHQ-D, Depression (Delta method standard errors, bias-corrected percentile bootstrap confidence intervals)*.

## Discussion

As its main objective, this study analyzed the use of coping strategies by the general population, with attention to sociodemographic variables, testing the capacity for mediation of these strategies in repercussions on mental health. Coping strategies focused on emotion, such as rumination, can be maladaptive, since the attempt to get more information on the dysphoric mood causes one to think repeatedly about the same thing, without attempting to solve the problem (13). Our results support the first hypothesis posed, since it was observed that maladaptive strategies such as rumination correlated negatively with age and marital status, where young adults and singles were those who most used this coping strategy. A study done in the USA during confinement of the population also found that young adults used less adaptive coping strategies (21).

The results also corroborate our second hypothesis, as men and women used different coping strategies. Women's means were higher in acceptance, rumination, positive refocusing and putting into perspective. And men scored significantly higher other-blame. These results are shared by other authors that have shown that women use more coping strategies focused on emotion, which could prevent depression, while men use more self-distraction and other-blame (20, 22).

Our results show that people use different coping strategies depending on their level of education as posed in Hypothesis 3. Those with a higher education use more rumination, planning, positive reappraisal and putting into perspective strategies. Individuals with a primary/secondary education scored higher in self-blame, catastrophizing and other-blame, coinciding with results found also by other authors (26).

It has been confirmed that home confinement due to health emergency has many effects on psychological well-being (2, 37–41). Our results are along this line, confirming our Hypothesis 4, as negative coping strategies, such as rumination and catastrophizing correlated positively with all the health subscales, while self-blame and other-blame strategies were positively related with the presence of somatic symptoms, anxiety/insomnia and depression. These results coincide with previous studies which reflected that negative coping strategies were related to health problems such as anxiety (5) and stress or depression (6).

Moreover, our results show that positive refocusing and reappraisal correlated negatively with anxiety/insomnia, social dysfunction and depression, and planning was negatively correlated to social dysfunction and depression. This is in agreement with the results of Guo et al. (9) and Goodarzi et al. (8) who observed that problem-focused coping was related to fewer health problems and greater well-being.

Similarly, the results of this study demonstrated that negative coping strategies exerted a mediating effect on the development of somatic symptoms, anxiety/insomnia and depression in those who had COVID-19 positive cases nearby. The mediating role of strategies such as rumination have already been described elsewhere (14). However, Model 2 shows that the use of positive strategies did not buffer the relationship between the presence of COVID-19 nearby and impact on health. Gruszczyńska and Rzeszutek (18) also described the relationship of positive reappraisal with well-being of persons is complex since they found well-being worsened with time. Therefore, the results of these coping strategies are not necessarily as unified and beneficial as supposed (19).

With these results we can discern that the use of certain coping strategies has a mediating role on the relationship between COVID-19 positive cases nearby and repercussions that it has on mental health as we proposed in the last hypothesis posed.

### Limitations and Future Research

The main limitation of our study is its cross-sectional design, which does not allow us to show how these variables behave over time. Future studies should have longitudinal designs that can show these. Another limitation refers to data collection, which was done using self-report questionnaires, and so there may have been subjective or reliability biases. The technological tools used for snowball sampling and to divulge the questionnaires and online collect the data may have conditioned the subjects who answered, and did not get to the whole population. So future studies could use other strategies for data collection to be able to access different populations.

### Practical Implications

The COVID-19 pandemic has implications for individual and collective health and emotional and social functioning of the population. In addition to providing health care, health services have to consider psychosocial needs. This study has relevant practical implications that should be considered for intervention in the health of the population in adverse situations such as those triggered by the COVID-19 public health emergency. Interventions should be performed on levels of individuals to institutions, including coping strategies that are postulated as beneficial for the health, and further, consider that they must be adapted to the confinement situation. These interventions would serve as preventive measures for health problems, helping people to develop a wide repertoire of healthy coping strategies.

## Conclusions

Adverse situations such as those experienced during the worldwide health emergency caused by the SARS-CoV-2 coronavirus cause people to make use of different coping strategies to endure them. These could facilitate the appearance of health problems or act as buffers for them. The rumination coping strategy was the one most used by young adults and by singles. “Negative” coping strategies exerted an indirect effect as mediators on the impact that the presence of COVID-19 cases nearby had on health, however, this effect was not observed for “positive” coping strategies. Based on these results, it is important to design plans to help the population develop coping strategies that enable them to remain healthy in the face of the consequences derived from COVID-19. And also offer an intervention to familiars of patients COVID-19, when the illness is detected and he must to initiate the confinement protocol or if he is hospitalized even.

## Data Availability Statement

The data that support the findings of this study are available from the corresponding author upon reasonable request.

## Ethics Statement

The studies involving human participants were reviewed and approved by University of Almeria Bioethics Committee (Ref. UALBIO2020/032). Written informed consent to participate in this study was provided by the participants' legal guardian/next of kin.

## Author Contributions

MP-F, MM, and EF-M contributed to the concept, design, analysis, and interpretation of the data. ÁM contributed to the technical details and manuscript preparation. MP-F, MM, and EF-M contributed to collecting the data. JG contributed to critically revising the manuscript for important intellectual content and the final approval of the version to be published. All authors accept and agree that the work is original, any methods and data presented are described accurately and honestly, and any relevant interests have been disclosed.

## Conflict of Interest

The authors declare that the research was conducted in the absence of any commercial or financial relationships that could be construed as a potential conflict of interest.
